# Canine vector-borne pathogens in semi-domesticated dogs residing in northern Cambodia

**DOI:** 10.1186/s13071-016-1552-z

**Published:** 2016-05-10

**Authors:** Tawin Inpankaew, Sze Fui Hii, Wissanuwat Chimnoi, Rebecca J. Traub

**Affiliations:** Department of Parasitology, Faculty of Veterinary Medicine, Kasetsart University, Bangkok, 10900 Thailand; Faculty of Veterinary and Agricultural Sciences, University of Melbourne, Parkville, Victoria 3052 Australia

**Keywords:** Canine, Vector-borne, Dogs, Cambodia, *Babesia*, *Ehrlichia*, *Dirofilaria*, PCR, *Mycoplasma*, *Hepatozoon*

## Abstract

**Background:**

In Southeast Asia, the canine vector-borne pathogens *Babesia* spp., *Ehrlichia canis, Anaplasma platys*, *Hepatozoon canis*, haemotropic mycoplasmas and *Dirofilaria immitis* cause significant morbidity and mortality in dogs. Moreover, dogs have also been implicated as natural reservoirs for *Rickettsia felis*, the agent of flea-borne spotted fever, increasingly implicated as a cause of undifferentiated fever in humans in Southeast Asia. The objective of this study was to determine the prevalence and diversity of canine vector-borne pathogens in 101 semi-domesticated dogs from rural Cambodia using molecular diagnostic techniques.

**Results:**

The most common canine vector-borne pathogens found infecting dogs in this study were *Babesia vogeli* (32.7 %) followed by *Ehrlichia canis* (21.8 %), *Dirofilaria immitis* (15.8 %), *Hepatozoon canis* (10.9 %), *Mycoplasma haemocanis* (9.9 %) and “*Candidatus* Mycoplasma haematoparvum” (2.9 %). A high level of co-infection with CVBD agents (23.8 %) was present, most commonly *B. vogeli* and *E. cani*s. Naturally occurring *R. felis* infection was also detected in 10.9 % of dogs in support of their role as a natural mammalian reservoir for flea-borne spotted fever in humans.

**Conclusions:**

This study reports for the first time, the prevalence and diversity of CVBD pathogens in dogs in Cambodia. In total, five species of CVBD pathogens were found infecting semi-domesticated dogs and many were co-infected with two or more pathogens. This study supports the role of dogs as natural mammalian reservoirs for *R. felis*, the agent of flea-borne spotted fever in humans.

## Background

In Southeast Asia, canine vector-borne diseases (CVBD) are a significant cause of morbidity and mortality in dogs. Dogs infected with vector-borne pathogens may develop either a sub-clinical or clinical infection, which may lead to fatal outcomes in some cases. In addition, dogs have also been implicated as natural mammalian reservoirs for *R. felis,* the agent of cat-flea-typhus or flea-borne spotted fever (FSF) in humans [1]. Despite the growing number of studies on the prevalence and distribution of CVBD in Asia, there is a paucity of information available on these agents in Cambodia.

In Southeast Asia, canine babesiosis caused by the piroplasms *Babesia vogeli* and *Babesia gibsoni* are vectored by *Rhipicephalus sanguineus* and *Haemaphysalis longicornis,* respectively*.* The latter may also be transmitted by blood exchange in fighting dogs [2] and vertically [3]. *Ehrlichia* is an alpha-proteobacterium genus belonging to the family Anaplasmataceae. Canine monocytic ehrlichiosis or tropical canine pancytopenia caused by *Ehrlichia canis* and vectored by *R. sanguineus* is widely distributed throughout Southeast Asia [4–6] and an important cause of mortality in dogs. Canine hepatozoonosis is a mild tick-borne protozoan disease caused by the apicomplexan parasite *Hepatozoon canis* in Asia*.* Transmission of *H. canis* to dogs occurs by ingestion of an infected *R. sanguineus* tick*,* rather than a tick bite [7]. Co-infection of *H. canis* with other infectious agents such as *Ehrlichia* and parvovirus is common [8, 9] and may contribute to or exacerbate clinical disease. Three species, *Mycoplasma haemocanis,* “*Candidatus* Mycoplasma haematoparvum” and “*Candidatus* Mycoplasma haemobos” have been reported in dogs and the clinical effects of these pathogens varies from asymptomatic to the stimulation of a hemolytic syndrome [10]. In Southeast Asia evidence to support the importance of *Anaplasma platys* as a cause of canine infectious cyclic thrombocytopenia is lacking, owing primarily to a paucity of published data [11, 12]. Single infections with *A. platys* are generally clinically unapparent but pathogenicity appears to be increased in co-infections [10]. Canine dirofilariosis is a mosquito-borne disease caused by *Dirofilaria immitis*. It is a chronic, progressive and potentially lethal disease whose primary lesions occur in the pulmonary arteries and lung parenchyma, leading to congestive heart failure [13].

*Rickettsia felis* is a globally emerging zoonotic pathogen responsible for flea-borne spotted fever (FSF) in humans. Worldwide, *Ctenocephalides felis* and in India, *Ctenocephalides felis orientis* have been implicated the most likely biological vectors for *R. felis* Cal2 and *R. felis*-like species RF2125, respectively. The agent is being increasingly implicated as an important cause non-specific febrile illness in humans in Laos [14], Bangladesh [15], Kenya [16] and Senegal [17]. In addition it may lead to severe multi-systemic disease owing to disseminated vasculitis [18–20]. Dogs [1, 21] and more recently cats [22] have been implicated as natural mammalian reservoirs for infection based on the detection of circulating *R. felis* DNA in peripheral blood.

The aim of this study was to investigate the occurrence of the primary CVBD of veterinary and public health importance in Cambodia using molecular diagnostic techniques.

## Methods

### Sampling and DNA extraction

**Fig 1.**
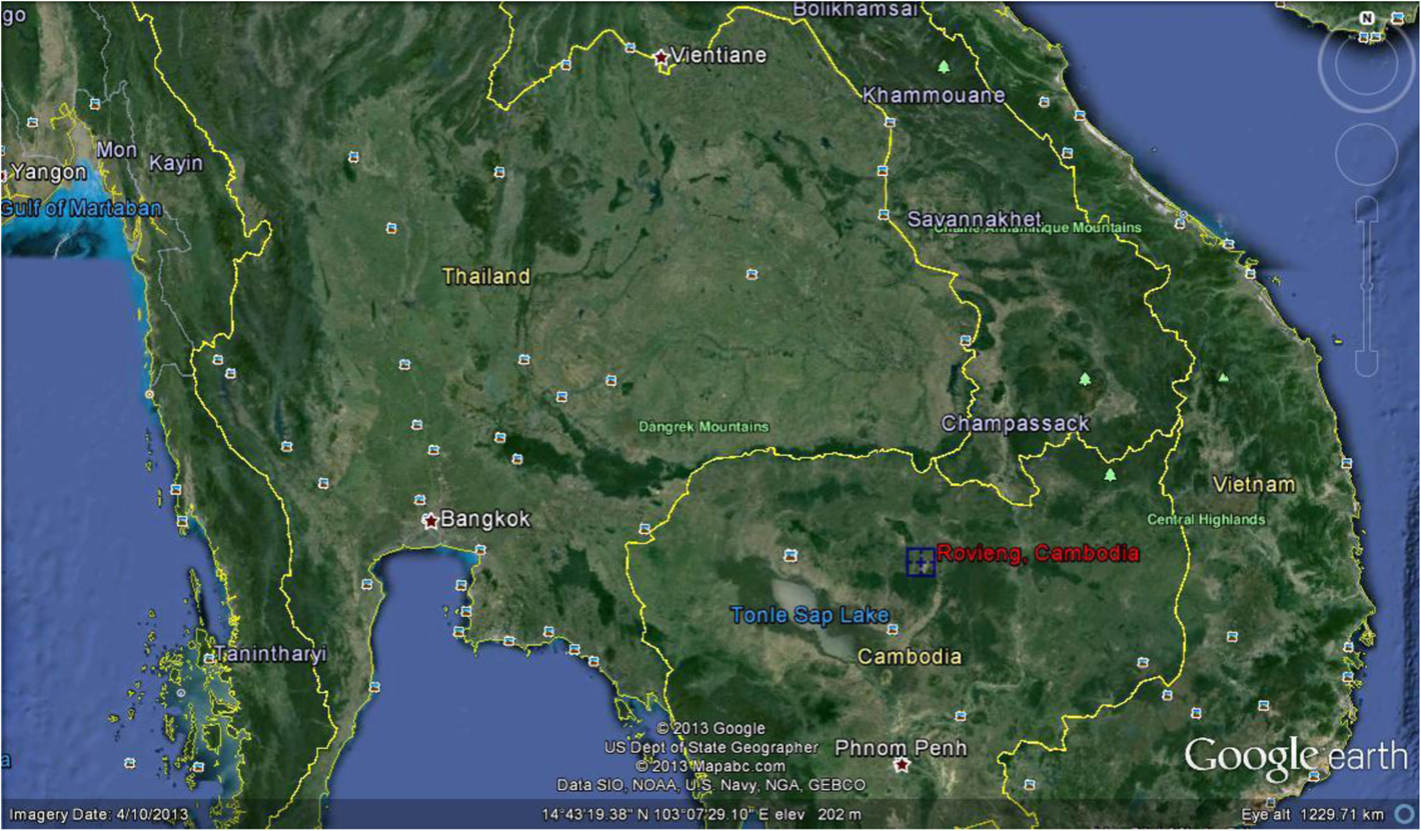
Map of Cambodia with Rovieng District, Preah Vihear Province highlighted in red. (Google Earth)

Blood samples were collected into sodium citrate blood tubes from 101 semi-domesticated, free-roaming dogs owned by residents belonging to 37 households in Dong Village, Rovieng district, Preah Vihear province, Cambodia (Fig. 1). Dogs in these communities lacked access to veterinary care, including vaccination, sterilisation, deworming and ectoparasite treatment. All blood samples were subjected to proteinase digestion in the presence of sodium dodecyl sulphate (SDS) followed by phenol-chloroform extraction of proteins and ethanol/salt precipitation of DNA as described elsewhere [23]. Final elution of DNA was made in 100 μl of TE buffer (10 mMTris-Cl, 0.5 mM EDTA, pH 9.0). The extracted DNA was stored at -20 °C until required for PCR amplification.

### PCR analysis and sequencing

All extracted DNA were subjected to previously described PCR assays for the detection of CVBD and spotted fever rickettsiae, as shown in Table 1. Samples positive for *R. felis* on the *omp*B gene were also subjected to a species-specific nested PCR targeting a 654 bp region of the *glt*A gene (Table 1). Genomic DNA of following organisms were used as positive controls for each PCR assay: *B. vogeli, A. platys, R. felis, M. haemocanis, H. canis, D. immitis* and *E. canis*. Nuclease-free water was used as a negative control. All the positive PCR products were purified using PureLink quick PCR purification kit (Invitrogen, Eugene, USA) according to the manufacturer’s protocol and submitted to the University of Queensland Animal Genetic Laboratory for DNA sequencing. All sequences were viewed using Finch TV 1.4.0 (Geospiza Inc.) and compared with known sequences from the GenBank™ database using the BLAST algorithm (http://blast.ncbi.nlm.nih.gov/Blast.cgi).

**Table 1:** Oligonucleotide primers used to detect vector borne protozoan and rickettsial pathogens

Pathogen	Specific	Gene target (bp)	Reference
*Babesia* spp.	Genus	18S rRNA (422–440)	[44]
*Hepatozoon* spp.	Genus	18S r RNA (666)	[45]
*Mycoplasma* spp.	Genus	16S rRNA (595)	[46]
*Ehrlichia* spp.	Genus	16S rRNA (345)	[47]
*A. platys*	Species	16S r RNA (678)	[47]
SFG rickettsiae	Group	*omp*B (252)	[48]
*R. felis*	Species	*glt*A (654)	[21]
*D. immitis*	Genus	5.8S-ITS2-28S (430–664)	[49]

### Phylogenetic analysis

DNA sequences of the partial *glt*A gene of samples positive for *R. felis* were viewed using Finch TV 1.4.0 (Geospiza, Inc.) and aligned using BioEdit version 7.2.0 [24] together with the *glt*A gene of the following rickettsiae species: *R. felis-*like species RF2125, *R. felis* Cal 2 strain, *R. australis*, *R. typhi*, *R. prowazekii*, *R. asiatica*, *R. conorii*, *R. rickettsia*, *R. honei*, *R. marmionii* (GenBank accession nos. AF516333, CP000053, U59718, U59714, M17149, AB297812, HM050292, DQ150689, AF018074, and AY737684, respectively). Neighbor-joining analyses were conducted with Tamura-Nei parameter distance estimates, and trees were constructed using Mega 6 software (www.megasoftware.net). Bootstrap analyses were conducted using 1000 replicates.

### Statistical analysis

Statistical analysis was conducted using STATA version 12.1 [25]. The association between CVBD agent positivity and the age and sex of dogs were evaluated using X^2^ test. Statistical significant was consider at *P*-value <0.05 with 95 % confidence interval (CI).

### Animal ethics

Written informed consent was obtained from each dog owner prior to sample collection. This study adhered to strict guidelines outlined by the European Convention for the “Protection of Vertebrate Animals used for Experimental and Other Scientific Purposes”. In addition, permission was gained from the Ministry of Agriculture, Forestry and Fisheries, Cambodia who were present for sampling and personally oversaw that animals were handled with respect according to the laws on experimental animal care in Cambodia.

## Results

### Detection of canine vector-borne pathogens

 Of 101 dogs sampled, 43 were male and 58 female. The sampled group consisted of 43 juveniles (12 weeks – 1 year), 56 adults (> 1–7 years) and 2 geriatric (> 7 years) dogs. Although a detailed physical examination was not conducted, all dogs were of mixed local breed and appeared bright and alert. In total, 72 dogs (71.3 %) were positive for at least one CVBD pathogen. DNA of *B. vogeli*, *E. canis*, *D. immitis*, *H. canis*, *M. haemocanis* and “*Candidatus* M. haematoparvum” was detected in 32.7 % (33/101), 21.8 % (22/101), 15.8 % (16/101), 10.9 % (11/101), 9.9 % (10/101) and 2.9 % (3/101) dogs, respectively (Table 2). Multiple infections were detected in 20 (19.8 %) of these infected dogs (Table 2). Additionally, one dog (0.9 %) was co-infected with four vector-borne pathogens in this study. In total, 10.9 % (11/101) dogs were found positive for *R. felis*. In three households, more than one dog was found harboring *R. felis* (Fig. 1).

**Table 2:** Prevalence of canine vector borne pathogens detected by PCR

Pathogen	Dogs (*n* = 101)
	Number positive (%)
*Babesia vogeli* (total)	33 (32.7)
*Ehrlichia canis* (total)	22 (21.8)
*Hepatozoon canis* (total)	11 (10.9)
*Anaplasma platys* (total)	0 (0.0)
*Mycoplasma haemocanis* (total)	10 (9.9)
*“Candidatus* Mycoplasma haematoparvum” (total)	3 (2.9)
*Dirofilaria immitis* (total)	16 (15.8)
*Rickettsia felis* (total)	11 (10.9)
*B. vogeli* and *M. haemocanis*	1 (0.9)
*B. vogeli* and “*Candidatus* Mycoplasma haematoparvum”	1 (0.9)
*B. vogeli* and *H. canis*	1 (0.9)
*B. vogeli* and *E. canis*	5 (4.9)
*B. vogeli* and *R. felis*	1 (0.9)
*B. vogeli* and *D. immitis*	1 (0.9)
*E. canis* and *R. felis*	2 (1.9)
*E. canis* and *M. haemocanis*	1 (0.9)
*H. canis* and *D. immitis*	1 (0.9)
*R. felis* and *D. immitis*	1 (0.9)
*B. vogeli*, *M. haemocanis* and *D. immitis*	1 (0.9)
*B. vogeli*, *E. canis* and *H. canis*	1 (0.9)
*B. vogeli*, *E. canis* and *R. felis*	2 (1.9)
*B. vogeli, E. canis* and *M. haemocanis*	1 (0.9)
*B. vogeli, E. canis* and *D. immitis*	2 (1.9)
*B. vogeli, H. canis* and *D. immitis*	1 (0.9)
*B. vogeli, H. canis, M. haemocanis* and *D. immitis*	1 (0.9)
Total infected dogs	72 (71.3)
Total co-infected dogs	24 (23.8)

### Phylogenetic analysis

**Fig. 2:**
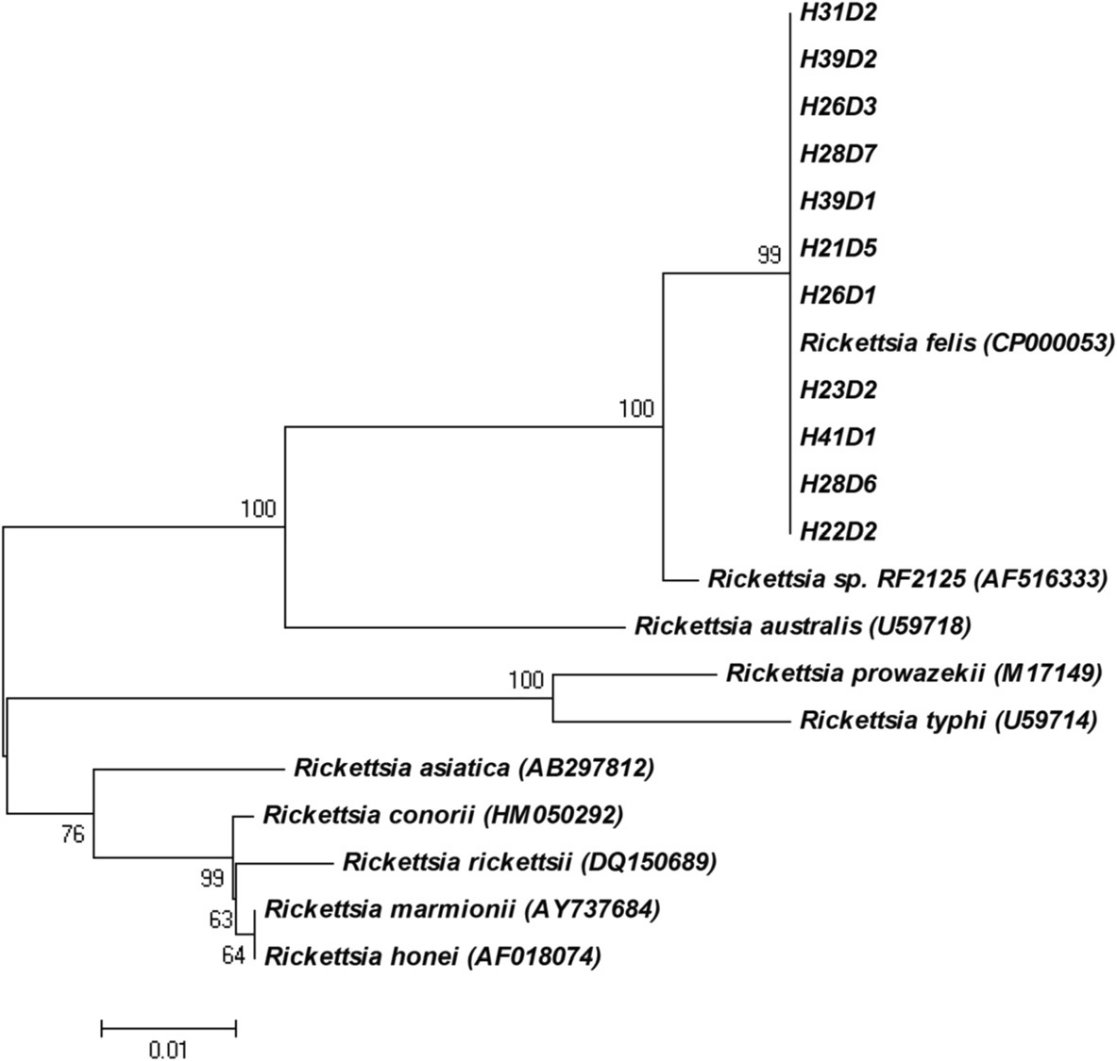
Phylogenetic tree obtained from neighbor-joining analysis of the *glt*A gene amplicons (600 bp) of *Rickettsia felis* detected in eleven dogs clustered with *R. felis* Cal 2 strain (CP000053)

DNA sequences of all positive PCR products were > 99–100 % homologous to those available in the GenBank™ database for all CVBD pathogens identified. Subsequent *R. felis*-specific nested PCR was successful for all eleven SFG-positive dogs. Resulting DNA sequences were > 99 % homologous to the *R. felis* Cal 2 strain on the *gltA* gene (GenBank: CP000053) (Fig. 2) and all eleven isolates clustered with *R. felis* Cal2 strain (99 % bootstrap support).

### Statistical analysis

There were no significant associations between the dogs’ sex and age and the presence of CVBD infections in this study.

## Discussion

This study represents the first report of CVBD infections in dogs from Cambodia. Our results indicate that CVBD pathogens *B. vogeli*, *E. canis*, *D. immitis*, *H. canis*, *M. haemocanis* and “*Candidatus* M. haematoparvum” are highly endemic in this area of Cambodia. Concurrent infection with two or more CVBD pathogens (19.8 %) was common among dogs and *B. vogeli* and *E. canis* constituted the most common dual infections. Co-infection with two or more CVBD pathogens is known to be associated with greater pathogenicity. Co-infection in dogs infected with tick-borne pathogens including *E. canis*, *B. vogeli*, *A. platys* and *H. canis* have been associated with a greater degree of anemia for example, than single infections [26]. This consequence has important implications on the health of dogs in these communities, although this clinical aspect was not further explored in this study. Our results also highlight the importance of screening for more than one pathogen in veterinary practice in Cambodia. Although not quantified, a high burden of ticks and flea infestation was observed on dogs during blood collection.

The absence of *A. platys* in this study is surprising. *A. platys* shares a common vector with all reported CVBD pathogens in this study and has been reported infecting 4/30 refuge dogs in nearby Malaysia and in 2.3 % of ticks in neighboring Thailand [11]. *Babesia gibsoni* has also been reported in dogs in nearby Malaysia at a rate of 17.1 % [27]. Numerous studies in neighboring Thailand, however, have not detected the pathogen in either ticks or dogs to date. In Thailand, this may be due to the reported absence to date of the tick vector *H. longicornis* [28, 29].

Canine heartworm or *D. immitis* is highly endemic in Southeast Asia with a reported prevalence ranging between 18.2 and 25.8 % in stray and client-owned dogs residing in Thailand, South Korea, China, Taiwan and Malaysia [30–34]. PCR assays can only detect DNA of circulating microfilaria in blood [35]. Therefore, the reported prevalence of heartworm infection in these community dogs of 15.8 % may be a gross underestimation. In heartworm endemic regions of India [36] and Australia [37], occult infections that have reported to comprise up to 30 % of infections and therefore future use of antigen-based tests would be of great advantage in gaining a more accurate estimate of infection levels in this community.

Nearly 11 % of dogs sampled in this study were positive for *R. felis*, adding evidence to suggest that dogs are the primary mammalian reservoir hosts for this emerging rickettsial zoonosis. The strain of *R. felis* detected in this study was 100 % homologous with the *R. felis* Cal 2 strain, hypothesized to have a co-evolutionary adaptive relationship with *C. felis felis* [38, 39]. In India and neighboring Thailand and Myanmar, *Rickettsia felis*-like species/strains, *R. felis-*like species RF2125 and *R. felis*-like species RF31 were detected in *C. felis orientis,* the most common flea species infesting dogs and cats in India and South East Asia [38, 40, 41]*.* Therefore, it is surprising that dogs in this Cambodian village harbor *R. felis* Cal 2 strain. Unfortunately, fleas were not sampled for species identification in this study, but future identification of fleas and the *R. felis*-like strains/species they harbor, will assist unravelling this hypothesis.

No significant risk factors were found associated with CVBD infections in this study. Of interest was that in three households, two of the resident dogs were found infected with *R. felis,* suggesting a household-level clustering effect. The finding of *R. felis* in this study suggests that dogs may act as a potential source of human rickettsial infection [42]. Scrub typhus and murine typhus have been reported in patients with unknown febrile illness in Cambodia [43]. However, since antibodies to murine typhus may cross-react with those of *R. felis*, it is plausible that some of these cases may be attributed to flea-borne spotted fever. Following the detection of *R. felis* in dogs in rural Cambodia, local health authorities should be made aware of the clinical consequences of FSF and include the agent as part of their routine diagnostic screening for illnesses ranging from undifferentiated febrile disease to those resembling spotted fevers/typhus.

## Conclusion

This study reports for the first time the prevalence and diversity of CVBD pathogens in dogs in Cambodia. In total five species of CVBD pathogens were found infecting semi-domesticated dogs and many were co-infected with two or more pathogens. This study supports the role of dogs as natural mammalian reservoirs for *R. felis*, the agent of flea-borne spotted fever in humans.

## References

[1] Hii SF, Kopp SR, Abdad MY, Thompson MF, O'Leary CA, Rees RL, Traub RJ (2011). Molecular evidence supports the role of dogs as potential reservoirs for *Rickettsia felis*. Vector-Borne Zoonotic Dis.

[2] Jefferies R, Ryan UM, Jardine J, Broughton DK, Robertson ID, Irwin PJ (2007). Blood, bull terriers and babesiosis: further evidence for direct transmission of *Babesia gibsoni* in dogs. Aust Vet J.

[3] Fukumoto S, Suzuki H, Igarashi I, Xuan X (2005). Fatal experimental transplacental *Babesia gibsoni* infections in dogs. Int J Parasit.

[4] Irwin PJ, Jefferies R (2004). Arthropod-transmitted diseases of companion animals in Southeast Asia. Trends Parasitol.

[5] Nazari M, Lim SY, Watanabe M, Sharma RSK, Cheng NABY, Watanabe M (2013). Molecular detection of *Ehrlichia canis* in dogs in Malaysia. PLoS Negl Trop Dis.

[6] Pinyoowong D, Jittapalapong S, Suksawat F, Stich RW, Thamchaipenet A (2008). Molecular characterization of Thai *Ehrlichia canis* and *Anaplasma platys* strains detected in dogs. Infect Genet Evol.

[7] Baneth G, Mathew JS, Shkap V, Macintire DK, Barta JR, Ewing SA (2003). Canine hepatozoonosis: two disease syndromes caused by separate *Hepatozoon* spp. Trends Parasitol.

[8] Baneth G, Aroch I, Presentey B (1997). *Hepatozoon canis* infection in a litter of Dalmatian dogs. Vet Parasitol.

[9] Baneth G, Harrus S, Gal A, Aroch I (2015). Canine vector-borne co-infections: *Ehrlichia canis* and *Hepatozoon canis* in the same host monocytes. Vet Parasitol.

[10] Hii S, Traub R, Thompson M, Henning J, O'Leary C, Burleigh A, McMahon S, Rees R, Kopp S (2015). Canine tick-borne pathogens and associated risk factors in dogs presenting with and without clinical signs consistent with tick-borne diseases in northern Australia. Aust Vet J.

[11] Foongladda S, Inthawong D, Kositanont U, Gaywee J (2011). Rickettsia, *Ehrlichia*, *Anaplasma*, and *Bartonella* in ticks and fleas from dogs and cats in Bangkok. Vector Borne Zoonotic Dis.

[12] Mokhtar AS, Lim SF, Tay ST (2013). Molecular detection of *Anaplasma platys* and *Babesia gibsoni* in dogs in Malaysia. Trop Biomed.

[13] McCall JW, Genchi C, Kramer LH, Guerrero J, Venco L (2008). Heartworm disease in animals and humans. Adv Parasitol.

[14] Dittrich S, Phommasone K, Anantatat T, Panyanivong P, Slesak G, Blacksell SD, Dubot-Peres A, Castonguay-Vanier J, Stenos J, Newton PN, Paris DH (2014). *Rickettsia felis* infections and Comorbid conditions, Laos, 2003–2011. Emerg Infect Dis.

[15] Ferdouse F, Hossain MA, Paul SK, Ahmed S, Mahmud MC, Ahmed R, Haque AK, Khan MN, Ghosh S, Urushibara N, Kobayashi N (2015). *Rickettsia felis* infection among humans, Bangladesh, 2012–2013. Emerg Infect Dis.

[16] Maina AN, Knobel DL, Jiang J, Halliday J, Feikin DR, Cleaveland S, Ng'ang'a Z, Junghae M, Breiman RF, Richards AL, Njenga MK (2012). *Rickettsia felis* infection in febrile patients, western Kenya, 2007–2010. Emerg Infect Dis.

[17] Socolovschi C, Mediannikov O, Sokhna C, Tall A, Diatta G, Bassene H, Trape JF, Raoult D (2010). *Rickettsia felis*-associated Uneruptive Fever, Senegal. Emerg Infect Dis.

[18] Lindblom A, Severinson K, Nilsson K (2010). *Rickettsia felis* infection in Sweden: report of two cases with subacute meningitis and review of the literature. Scand J Infect Dis.

[19] Williams M, Izzard L, Graves SR, Stenos J, Kelly JJ (2011). First probable Australian cases of human infection with *Rickettsia felis* (cat-flea typhus). Med J Aust.

[20] Zavala-Castro J, Zavala-Velazquez J, Walker D, Perez-Osorio J, Peniche-Lara G (2009). Severe human infection with *Rickettsia felis* associated with hepatitis in Yucatan, Mexico. Int J Med Microbiol.

[21] Hii SF, Kopp SR, Thompson MF, O'Leary CA, Rees RL, Traub RJ (2011). Molecular evidence of *Rickettsia felis* infection in dogs from northern territory, Australia. Parasit Vectors.

[22] Ahmed R, Paul SK, Hossain MA, Ahmed S, Mahmud MC, Nasreen SA, Ferdouse F, Sharmi RH, Ahamed F, Ghosh S, Urushibara N, Aung MS, Kobayashi N (2016). Molecular detection of *Rickettsia felis* in humans, cats, and cat fleas in Bangladesh, 2013–2014. Vector Borne Zoonotic Dis.

[23] Bremer WG, Schaefer JJ, Wagner ER, Ewing SA, Rikihisa Y, Needham GR, Jittapalapong S, Moore DL, Stich RW (2005). Transstadial and intrastadial experimental transmission of *Ehrlichia canis* by male *Rhipicephalus sanguineus*. Vet Parasitol.

[24] Hall TA (1999). BioEdit: a user-friendly biological sequence alignment editor and analysis program for Windows 95/98/NT. Nucl Acids SympSer.

[25] StataCorp (2011). Stata statistical software: release 12.

[26] Rojas A, Rojas D, Montenegro V, Gutiérrez R, Yasur-Landau D, Baneth G (2014). Vector-borne pathogens in dogs from Costa Rica: first molecular description of *Babesia vogeli* and *Hepatozoon canis* infections with a high prevalence of monocytic ehrlichiosis and the manifestations of co-infection. Vet Parasitol.

[27] Rajamanickam C, Wiesenhutter E, Zin FM, Hamid J (1985). The incidence of canine haematozoa in Peninsular Malaysia. Vet Parasitol.

[28] Parola P, Sanogo OY, Lerdthusnee K, Zeaiter Z, Chauvancy G, Gonzalez JP, Miller RS, Telford SR, Wongsrichanalai C, Raoult D (2003). Identification of *Rickettsia* spp. and *Bartonella* spp. in fleas from the Thai-Myanmar border. Ann N Y AcadSci.

[29] Tanskull P, Inlao I (1989). Keys to the adult ticks of *Haemaphysalis* Koch, 1844, in Thailand with notes on changes in taxonomy (Acari: Ixodoidea: Ixodidae). J Med Entomol.

[30] Boonyapakorn C, Srikitjakarn L, Morakote N, Hoerchner F (2008). The epidemiology of *Dirofilaria immitis* infection in outpatient dogs at Chiang Mai University Small Animal Hospital, Thailand. Southeast Asian J Trop Med Public Health.

[31] Song KH, Park JE, Lee DH, Lee SH, Shin HJ (2010). Serological update and molecular characterization of *Dirofilaria immitis* in dogs. South Korea Res Vet Sci.

[32] Hou H, Shen G, Wu W, Gong P, Liu Q, You J, Cai Y, Li J, Zhang X (2011). Prevalence of *Dirofilaria immitis* infection in dogs from Dandong, China. Vet Parasitol.

[33] Wu CC, Fan PC (2003). Prevalence of canine dirofilariasis in Taiwan. J Helminthol.

[34] Retnasabapathy A, San KT (1976). Incidence of canine heartworm (*Dirofilaria immitis*) in Malaysia. Vet Rec.

[35] Duscher G, Peschke R, Wille-Piazzai W, Joachim A (2009). Parasites on paper--The use of FTA Elute ^(R)^ for the detection of *Dirofilaria repens* microfilariae in canine blood. Vet Parasitol.

[36] Borthakur SK, Deka DK, Islam S, Sarma DK, Sarmah PC (2015). Prevalence and molecular epidemiological data on *Dirofilaria immitis* in dogs from Northeastern States of India. Sci World J.

[37] Atwell R (1989). Heartworm disease and research in Australia. Br Vet J.

[38] Hii SF, Lawrence AL, Cuttell L, Tynas R, Abd Rani PAM, Slapeta J, Traub RJ (2015). Evidence for a specific host-endosymbiont relationship between '*Rickettsia* sp genotype RF2125' and *Ctenocephalides felis orientis* infesting dogs in India. Parasit Vectors.

[39] Lawrence AL, Hii SF, Jirsova D, Panakova L, Ionica AM, Gilchrist K, Modry D, Mihalca AD, Webb CE, Traub RJ, Slapeta J (2015). Integrated morphological and molecular identification of cat fleas (*Ctenocephalides felis*) and dog fleas (*Ctenocephalides canis*) vectoring *Rickettsia felis* in central Europe. Vet Parasitol.

[40] Changbunjong T, Buddhirongawatr R, Suwanpakdee S, Siengsanan J, Yongyuttawichai P, Cheewajorn K, Jangjaras J, Sangloung C, Ratanakorn P (2009). A survey of ectoparasitic arthropods on domestic animals in Tak province, Thailand. Southeast Asian J Trop Med Public Health.

[41] Kernif T, Socolovschi C, Wells K, Lakim MB, Inthalad S, Slesak G, Boudebouch N, Beaucournu JC, Newton PN, Raoult D, Parola P (2012). *Bartonella* and *Rickettsia* in arthropods from the Lao PDR and from Borneo, Malaysia. Comp Immunol Microb.

[42] Hii SF, Abdad MY, Kopp SR, Stenos J, Rees RL, Traub RJ (2013). Seroprevalence and risk factors for *Rickettsia felis* exposure in dogs from Southeast Queensland and the Northern Territory, Australia. Parasit Vectors.

[43] Kasper MR, Blair PJ, Touch S, Sokhal B, Yasuda CY, Williams M, Richards AL, Burgess TH, Wierzba TF, Putnam SD (2012). Infectious etiologies of acute febrile illness among patients seeking health care in South-Central Cambodia. Am J Trop Med Hyg.

[44] Földvári G, Hell E, Farkas R (2005). *Babesia canis canis* in dogs from Hungary: detection by PCR and sequencing. Vet Parasitol.

[45] Inokuma H, Okuda M, Ohno K, Shimoda K, Onishi T (2002). Analysis of the 18S rRNA gene sequence of a *Hepatozoon* detected in two Japanese dogs. Vet Parasitol.

[46] Criado-Fornelio A, Martinez-Marcos A, Buling-Saraña A, Barba-Carretero JC (2003). Presence of *Mycoplasma haemofelis*, *Mycoplasma haemominutum* and piroplasmids in cats from southern Europe: a molecular study. Vet Microbiol.

[47] Brown GK, Martin AR, Roberts TK, Aitken RJ (2001). Detection of *Ehrlichia platys* in dogs in Australia. Aust Vet J.

[48] Paris DH, Blacksell SD, Stenos J, Graves SR, Unsworth NB, Phetsouvanh R, Newton PN, Day NP (2008). Real-time multiplex PCR assay for detection and differentiation of rickettsiae and orientiae. Trans R Soc Trop Med Hyg.

[49] Rishniw M, Barr SC, Simpson KW, Frongillo MF, Franz M, Dominguez Alpizar JL (2006). Discrimination between six species of canine microfilariae by a single polymerase chain reaction. Vet Parasitol.

